# CASCADE: a novel quasi all paths-based network analysis algorithm for clustering biological interactions

**DOI:** 10.1186/1471-2105-9-64

**Published:** 2008-01-29

**Authors:** Woochang Hwang, Young-Rae Cho, Aidong Zhang, Murali Ramanathan

**Affiliations:** 1 Department of Computer Science and Engineering, State University of New York, Buffalo, NY 14260, USA; 2 Department of Pharmaceutical Sciences, State University of New York, Buffalo, NY 14260, USA

## Abstract

**Background:**

Quantitative characterization of the topological characteristics of protein-protein interaction (PPI) networks can enable the elucidation of biological functional modules. Here, we present a novel clustering methodology for PPI networks wherein the biological and topological influence of each protein on other proteins is modeled using the probability distribution that the series of interactions necessary to link a pair of distant proteins in the network occur within a time constant (the occurrence probability).

**Results:**

CASCADE selects representative nodes for each cluster and iteratively refines clusters based on a combination of the occurrence probability and graph topology between every protein pair. The CASCADE approach is compared to nine competing approaches. The clusters obtained by each technique are compared for enrichment of biological function. CASCADE generates larger clusters and the clusters identified have *p*-values for biological function that are approximately 1000-fold better than the other methods on the yeast PPI network dataset. An important strength of CASCADE is that the percentage of proteins that are discarded to create clusters is much lower than the other approaches which have an average discard rate of 45% on the yeast protein-protein interaction network.

**Conclusion:**

CASCADE is effective at detecting biologically relevant clusters of interactions.

## Background

Protein-protein interactions (PPI) and other biological interactions regulate a wide array of biological processes. In recent years, biophysical and biochemical approaches for PPI characterization have been supplemented by techniques such as the yeast two-hybrid and mass spectrometry, which have allowed large-scale characterization of a large number of PPIs [1-5]. Systematic analysis of the underlying relationships in PPI data sets can potentially provide useful insights into roles of proteins in biological processes [6].

The primary physicochemical determinants of the extent and rate of bimolecular PPIs are the equilibrium dissociation constant, the rate constant, reaction stoichiometry and the concentrations of free and bound interacting species. However, because of the limitations of existing experimental methods, the currently available PPI data sets are binary valued adjacency matrices that simply indicate whether or not two proteins interact under the assay conditions.

In cells, proteins usually function by interacting with other proteins either in pairs or as components of larger complexes. However, it is still difficult to obtain an accurate understanding of the functional modules, that encompass the groups of proteins involved in common elementary biological functions. A functional module can be defined as a set of proteins that together are involved in a biological process [7]. Hartwell et al. [6] defined a notion of a functional modules more generally as a group of cellular components and their interaction that can be attributed a specific biological function. Cluster analysis is the partitioning of a data set into subsets (clusters), so that the data in each subset share some common feature and can be grouped in the specific context of PPI networks, as proteins that share some biological/topological property. Cluster analysis is thus generally the method of choice for functional module detection, enabling a better understanding of topological structures and the relationships between components of a network.

### Related work

The binary nature of the current PPI data sets imposes challenges in clustering using conventional similarity or distance-based approaches that are effective in pattern recognition. For example, the reciprocal of the shortest path length and the hitting time for a random walk between two molecular components have been investigated as a distance/similarity measure for distance based clustering [8,9]. Iterative methods that employ shortest path calculations with hierarchical clustering to obtain distance/similarity measures method have also been investigated [8]. Many different clustering methods that integrate other biological information sources, e.g., Gene Ontology (GO), phylogenetic profiles, ortholog information, and gene expression, have been proposed to complement binary PPI data sets. Wu et al. integrated GO, phylogenetic profiles and gene neighbors using Bayesian inference to detect functional modules [10]. Snel et al. identified functional modules by selecting "linker" protein located between clusters of orthologous groups built from a comparative analysis of multiple genomes [7]. Tornow et al. integrated PPI networks and gene expression data to identify functional modules using the superparamagnetic clustering method (SPC) [11,12]. The modified betweenness cut approach has been proposed on weighted PPI networks that likewise, combined gene expression [13].

The PPI and biological interaction adjacency matrices can also be represented as graphs whose nodes represent molecular components and edges represent interactions. The clustering of a biological interaction dataset can be thereby reduced to graph theoretical problems. In the maximal clique approach, clustering is reduced to identifying fully connected subgraphs in the graph [14]. To overcome the relatively high stringency imposed by the maximal clique method, the Quasi Clique [15], Molecular Complex Detection (MCODE) [16], Spirin and Mirny [14] algorithms identify densely connected subgraphs rather than fully connected ones either by optimizing an objective density function or by using a density threshold. The Restricted Neighborhood Search Clustering Algorithm (RNSC) [17] and Highly Connected Subgraphs (HCS) algorithms [18] harness minimum cost edge cuts for cluster identification. The Markov Cluster Algorithm (MCL) algorithm finds clusters using iterative rounds of expansion and inflation that promote the strongly connected regions and weaken the sparsely connected regions, respectively [19]. The line graph generation approach [20] transforms the network of molecular components connected by interactions into a network of connected interactions and then uses the MCL algorithm to cluster the interaction network.

However, currently used graph theoretical approaches also encounter challenges because the relationship between protein function and PPI is characterized by weak connectivity. Indeed, most of the proteins annotated as being involved in a same function do not have direct physical interaction between them in a PPI network. For instance, we estimated the density of intra-connectivity of the 3rd level or more specific function within MIPS functional hierarchy: on average, only 8.7% of the possible connections within a 3rd or more specific function occur (11.0% of the possible connections within a 4th or more specific function occur) [21]. Therefore, An excessive emphasis on high connectivity can limit performance due to a bias for relatively balanced, round shaped clusters and produce a large number of unassigned proteins. In another direction, statistical approaches to clustering have also been proposed. For example, Samanta and Liang [22] employed a statistical approach to clustering of proteins based on the premise that a pair of proteins sharing a significantly larger number of common neighbors will have high functional similarity.

In this paper, we extend our earlier approach (STM) [23]. In STM, we modeled the biological and topological influence of each protein on other proteins in a protein network using the probability distribution that the series of interactions necessary to link a pair of distant proteins in the network occur within a time constant, i.e., the occurrence probability (see page 13). STM propagated the occurrence probability through a shortest path between a protein pair. However, in CASCADE, the occurrence probability of a series of pairwise interactions is propagated through the interaction network via the Quasi all paths (QAP) algorithm (see page 13 and Appendix), which approximates the all possible paths enumeration. CASCADE, is an enhanced effective novel clustering model and its QAP extension enables it to outperform the shortest path approach in STM.

The CASCADE algorithm can effectively detect both densely and sparsely connected, biologically relevant functional modules with few discards. We have compared CASCADE to competing approaches including STM and the results obtained demonstrate the superiority of the CASCADE strategy. The improvements in CASCADE, which include a refinement to the occurrence probability quantification function and an application of novel Quasi All Paths (QAP) method to incorporate network topology, enhanced its performance over STM on *p*-values for biological function by 76-fold on average.

## Results

### Analysis of Prototypical Data

**Figure 1:**
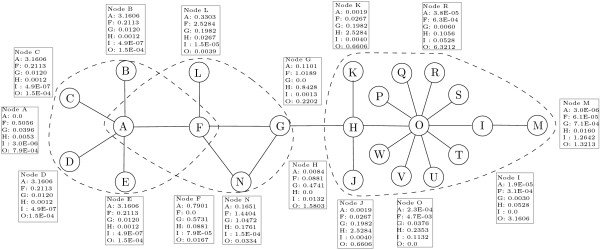
**A simple network**. Each box contains the numerical values obtained from Equation 2 from nodes *A*, *F*, *G*, *H*, *I *and *O *to other target nodes. The values for nodes *P*, *Q*, *S*, *T*, *U*, *V *and *W *are the same as node *R*'s. Results for other nodes are not shown. Final identified clusters are delimited when the merging threshold 2.0 is used.

To illustrate the principles underling the CASCADE approach, we first present the results from the analysis of the simple network shown in Figure 1. Briefly, the CASCADE algorithm involves four sequential processes:

#### Process 1

Propagate the occurrence probability from each node to the other nodes through Quasi All Paths in the network.

#### Process 2

Select cluster representatives for each node based on the accumulated occurrence probability quantity on each node.

#### Process 3

Preliminary clusters will be formed by aggregating each node into the clusters that the selected representatives have formed.

#### Process 4

Preliminary clusters will be merged if they have substantial similarity, i.e., inter-connectivity.

**Figure 2:**
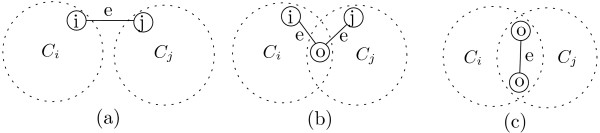
**Inter-connectivity**. Inter-connections between a cluster pair. (a) interconnecting edge *e *between two non-overlapping nodes (b) interconnecting edge *e *between an overlapping node and a non-overlapping node (c) interconnecting edge *e *between two overlapping nodes

First, the occurrence probability from each node will be propagated to the other nodes through QAPs in the network. Only the occurrence probability from nodes A, F, G, H, I and O are presented for ease of understanding in Figure 1. Each box in Figure 1 contains the weighted occurrence probability assessed by the Equation 2 (see Methods) from nodes A, F, G, H, I and O to other target nodes. These numerical values illustrate overall effects of combining the network topology with the occurrence probability quantification model. Second, the nodes selected as representatives during the second step are those with the highest values of the weighted occurrence probability. For example, nodes B, C, D, E, and F will choose node A and nodes A, G, L, and N will choose node F, which are the best scored nodes on those nodes, as their representatives. Third, preliminary clusters will be formed by accumulating each node toward their selected representatives. For example, in Figure 1, four preliminary clusters, *C*1 = {A, B, C, D, E, F}, *C*2 = {A, F, G, L, N}, *C*3 = {H, O, J, K}, and *C*4 = {I, H, M, O, P, Q, R, S, T, U, V, W}, are formed based on the choice of representatives. For the last step of CASCADE, preliminary clusters are merged if they have significant interconnections. Our definition of similarity between two clusters in Figure 2 and in Equation 3 (see Methods) counts various types of inter-connections, interconnecting edges between two non-overlapping nodes, interconnecting edges between an overlapping node and a non-overlapping node, interconnecting edges between two overlapping nodes. For example, a cluster pair that has an overlapping node having many edges in each cluster should have high similarity. For example in Figure 1, *C*3 and *C*4 has a common node *O *that has one edge in *C*3 and ten edges in *C*4. There are a total of ten inter-connecting edges for the cluster pair *C*3 and *C*4 since the edge between H and O is redundant. So, the similarity of each cluster pair will be follows: Similarity(*C*3, *C*4) = 10/4, Similarity(*C*1, *C*2) = 8/5, Similarity(*C*2, *C*3) = 1/4. Therefore, only one merge occurred between the cluster *C*3 and *C*4 because it is the only cluster pair with sufficient similarity for a merge threshold of 2.0. Eventually, two clusters, {A, B, C, D, E, F, G, L, N}, {H, I, J, K, M, O, P, Q, R, S, T, U, V, W}, are obtained after the merge process using 1.0 as the merge threshold. Three clusters, {A, B, C, D, E, F}, {A, F, G, L, N}, and {H, I, J, K, M, O, P, Q, R, S, T, U, V, W} are obtained and delimited in Figure 1 when 2.0 is used as the merge threshold.

### Significance of Individual Clusters

The characteristics of all 50 clusters with 5 or more proteins identified in the DIP yeast PPI network [24] using CASCADE are summarized in Additional file 1. It also shows the topological characteristics and their assigned molecular functions (the most commonly matched function category from the MIPS functional categories database was assigned to the cluster) for each cluster. To facilitate critical assessments, the percentage of proteins that are in concordance with the major assigned function (hits), the discordant proteins (misses) and unknown are also indicated.

The largest cluster in Additional file 1 contains 411 proteins and the smallest cluster contains 5. There are 48.1 proteins in a cluster on average and the average density of the subgraphs of the clusters extracted from the yeast core PPI network is 0.256. The -*log p *values of the major function identified in each cluster are also shown and these values provide a measure of the relative enrichment of a cluster for a given functional category: higher values of -*log p *indicate greater enrichment. The results demonstrate that the CASCADE method can detect large but sparsely connected clusters as well as small densely connected clusters. The high values of -*log p *(values greater than 2 indicate statistical significance at < 0.01) indicate that clusters are significantly enriched for biological function and can be considered to be functional modules.

Table 1 summarizes the characteristics of all clusters with 3 or more nodes detected by CASCADE on 3 biological network data sets (the yeast DNA damage response network, Rapamycin gene modules network, Rich medium gene modules network). It confirms that CASCADE can detect large but sparsely connected clusters as well as small densely connected clusters for a range of diverse data sets. Furthermore, the clusters identified are enriched for certain biological functions and may be considered to be functional modules.

**Table 1:** Clusters obtained using CASCADE for 3 biological network data sets (the yeast DNA damage response network, Ra-pamycin gene modules network, Rich medium gene modules network).

				Distribution				
						
Data set	Cluster	Size	Density	H	D	U	-Log *p*	Function
Yeast DDR network	1	49	0.063	18.4	81.6	0.0	0.5	DNA repair
	2	16	0.175	81.3	18.7	0.0	3.6	Cell cycle
	3	9	0.222	44.4	55.5	0.0	3.6	Proteasome
	4	7	0.286	57.1	42.9	0.0	1.7	Metabolism
	5	7	0.286	71.4	28.6	0.0	1.2	Stress response
	6	6	0.333	83.3	16.7	0.0	3.2	Metabolism
Rapamycin gene modules network	1	19	0.198	42.1	47.4	10.5	2.7	Nitrogen/sulfur metabolism
	2	12	0.227	33.3	0.0	66.6	1.1	Pheromone response
	3	9	0.277	77.8	0.0	22.2	5.0	Pheromone response
	4	7	0.285	71.4	28.6	0.0	2.9	AA metabolism/biosynthesis
Rich medium gene modules network	1	54	0.050	64.8	33.3	1.85	14.1	Cell cycle
	2	28	0.111	75.0	14.3	10.7	10.2	Ribosome biogenesis
	3	16	0.179	62.5	12.5	25.0	9.7	Respiration
	4	13	0.222	69.2	30.8	0.0	8.1	Energy/carbohydrate metabolism

The first column is a cluster identifier; the Size column indicates the number of molecular components in each cluster; the Density indicates the percentage of possible biological interactions that are present; the *H *column indicates the percentage of molecular components concordant with the major function indicated in the last column; the *D *column indicates the percentage of molecular components discordant with the major function and *U *column indicates percentage of molecular components not assigned to any function. The -log *p *values for biological function are shown.

### Analysis Of Functional Annotation

In order to scrutinize the functional term distribution of each detected cluster by CASCADE, the normalized number of the MIPS functional terms and the number of proteins that are associated with the MIPS functional terms in each cluster were analyzed.

Additional file 2 assesses the heterogeneity of functional terms from the MIPS database for each cluster detected by CASCADE. The results show that the clusters have high level of functional homogeneity even after correcting for cluster size.

Additional file 3 summarizes the MIPS functional categories for proteins in the ten largest clusters identified by CASCADE. Within each cluster, there was considerable functional homogeneity as assessed by the relatedness among functional categories, e.g., Cluster 3 was enriched for RNA transport processes. Furthermore, as would be expected, the largest clusters also contained certain general functions that are required for numerous cellular process, e.g., mRNA synthesis was present in Clusters 1, 2 and 3.

The results from Additional file 4, 5, 6, 7 show that the densities of the subgraphs for each cluster in the PPI network is low and that the topological shapes are diverse. Despite the low density and variable shape, CASCADE was found to identify and assign a high proportion of proteins to the dominant functional category. For example in Additional file 6, CASCADE detected the cluster containing protein YIR009W, YPL213W, and YNR011C and they have very good functional homogeneity with other members in the cluster. The performance of competing approaches was affected adversely by weak connectivity.

### Comparative Assessment

To demonstrate the strengths of the CASCADE approach, we compared it to the following ten competing clustering approaches: Maximal clique [14], Quasi clique [15], Minimum cut [25], Betweenness cut [26], the statistical approach of Samanta and Liang [22], MCL [19], Chen [13], Rives [8], SPC [11], and STM [23]. The results for clusters are summarized in Table 2 and 3. The -log *p *values in Table 2 and 3 are the average -log *p *values of all detected clusters by each method.

**Table 2:** Comparison of CASCADE to competing clustering methods for 2 biological network data sets (BIOGRID Yeast PPI network, DIP Yeast PPI network).

Dataset	Method	Cluster	Size	Discard	MIPS (-log*p*)			GO (-log*p*)		
		Number		(%)	Function	Location	Complex	mf	cc	bp
BIOGRID Yeast PPI network	**CASCADE**	**225**	**19.6**	**18.3**	**3.26**	**2.55**	**5.13**	**4.67**	**4.24**	**3.53**
	STM	248	18.1	16.2	2.88	2.37	4.64	4.17	3.98	3.53
	Maximal clique	587	3.6	80.8	2.71	2.21	4.53	3.55	3.47	2.99
	Quasi clique	431	7.4	40.9	2.97	2.03	4.89	4.16	3.88	3.02
	Samanta	289	6.7	64.8	2.63	1.61	4.59	3.48	3.29	3.01
	MCL	617	6.2	29.2	2.58	1.22	3.87	4.02	3.77	2.83
	Chen	577	8.4	10.1	2.61	2.08	4.13	4.36	3.84	3.05
	Rives	217	21.5	13.5	3.04	2.34	4.22	4.14	3.97	3.03
	SPC	85	54.9	13.4	1.33	0.87	2.65	2.11	2.51	2.29

DIP Yeast PPI network	**CASCADE**	**50**	**48.1**	**7.3**	**14.1**	**7.84**	**15.8**	**12.1**	**12.8**	**9.09**
	STM	60	40.1	7.8	13.0	7.23	14.2	11.8	11.9	8.04
	Maximal clique	120	5.7	98.3	10.2	7.67	10.0	8.46	10.0	6.57
	Quasi clique	103	11.2	80.8	11.0	6.29	12.0	10.7	11.1	7.69
	Samanta	64	7.9	79.9	8.76	4.74	10.7	9.82	10.8	8.01
	Minimum cut	114	13.5	35.0	7.97	4.58	8.56	8.19	7.87	6.21
	Betweenness cut	180	10.3	21.0	7.89	4.06	8.59	7.02	6.98	4.88
	MCL	163	9.8	36.7	8.08	3.84	9.53	7.81	8.11	6.26
	Chen	141	16.3	1.7	9.12	4.91	9.87	8.28	8.09	6.01
	Rives	42	55.3	7.8	10.1	6.88	9.52	9.61	9.59	7.42
	SPC	5	47.2	6.4	5.27	2.39	5.49	6.23	5.91	5.18

The Number column indicates the number of clusters identified by each method, the Size column indicates the average number of molecular components in each cluster; the Discard(%) indicates the percentage of molecular components not assigned to any cluster. The average -log *p *values of all detected clusters for MIPS categories (biological function, cellular location, complex) and Gene Ontology (molecular functions (mf), biological process (bp), cellular component (cc)) are shown. Comparisons are performed on the clusters with 5 or more molecular components. The results for Minimum cut and Betweenness cut for the BIOGRID dataset are not shown due to limitation of the available implementation.

**Table 3:** Comparison of CASCADE to competing clustering methods for 3 biological network data sets (Yeast DNA damage response network, Rapamycin gene modules network, and Rich medium gene modules network).

Dataset	Method	Number	Size	Discard(%)	Function (-log *p*)
DNA damage response network	**CASCADE**	**6**	**15.7**	**5.0**	**2.28**
	STM	6	16.0	5.2	2.28
	Quasi clique	3	7.0	88.5	0.87
	Samanta	6	6.7	58.3	1.79
	Minimum cut	7	13.1	4.2	1.18
	Betweenness cut	10	8.8	8.3	2.22
	MCL	3	9.3	70.8	2.37
	Chen	7	13.7	0.0	2.66
	Rives	5	18.4	4.1	1.61
	SPC	3	20.3	36.5	2.33

Rapamycin gene modules network	**CASCADE**	**4**	**11.8**	**6.0**	**2.90**
	STM	4	12.5	0.0	2.57
	Quasi clique	13	8.2	0.0	2.17
	Samanta	7	4.9	32.0	1.57
	Minimum cut	8	5.9	6.0	1.82
	Betweenness cut	5	8.0	20.0	2.03
	MCL	6	7.7	8.0	5.48
	Chen	5	10.0	0.0	2.01
	Rives	4	11.0	12.0	1.49
	SPC	3	15.3	8.0	1.47

Rich medium gene modules network	**CASCADE**	**4**	**27.8**	**0.0**	**10.5**
	STM	5	22.4	0.0	8.21
	Quasi clique	5	22.8	0.0	7.81
	Samanta	12	5.3	43.2	4.79
	Minimum cut	10	11.1	0.0	4.41
	Betweenness cut	8	13.9	0.0	6.38
	MCL	23	4.0	4.5	7.29
	Chen	8	13.9	0.0	6.13
	Rives	5	22.2	0.0	5.77
	SPC	5	20.6	7.2	6.80

The Number column indicates the number of clusters identified by each method, the Size column indicates the average number of molecular components in each cluster; the Discard(%) indicates the percentage of molecular components not assigned to any cluster. The average -log *p *values of all detected clusters for biological function are shown. Comparisons are performed on the clusters with 5 or more molecular components for the first data sets(DNA damage response network), and on the clusters with 3 or more molecular components for the last 2 network data sets(Rapamycin gene modules networks, Rich medium gene modules network). The Maximal clique method is not included in the Table because none of the identified clusters have 3 or more members.

The experimental results for the BIOGRID PPI dataset [27] are presented in Table 2. The performance was measured for each MIPS and Gene Ontology category. Table 2 shows that CASCADE had lower *p*-values and outperformed the other methods on each MIPS and Gene Ontology category. On MIPS functional category, the clusters identified by CASCADE have *p*-values that are approximately 2.8-fold and 1.9-fold lower than STM and Rives approach, respectively, the best performing alternative clustering methods. On MIPS Localization category, CASCADE identified the clusters with *p*-values that are approximately 1.7-fold and 2.1-fold lower than STM and Rives approach, respectively. On MIPS complex category, the clusters detected by CASCADE have *p*-values that are approximately 5-fold and 3.4-fold lower than STM and Quasi clique approach, respectively. Similarly, CASCADE was also found to be superior with the Gene Ontology categories. Another important strength of CASCADE (and STM) method is that the percentage of proteins that are discarded to create clusters is 18.3%, which is much lower than the other approaches, which have an average discard rate of 33%.

The results in Table 2 for the DIP yeast PPI dataset [24] show that CASCADE generates larger clusters; the clusters identified have *p*-values on MIPS functional category that are approximately 6.3-fold and 1000-fold lower than STM and Quasi clique, respectively, the best performing alternative clustering methods. The *p*-values for cellular localization for CASCADE are comparable to those from the maximal clique method. In MIPS complex category, CASCADE showed the best *p*-values over STM and Quasi clique, the best performing alternative clustering methods. CASCADE (and STM) method discarded only 7.3% to identify clusters, which is much lower than the other approaches, which have an average discard rate of 45%. We also conducted these analyses for clusters with more than 9 members and obtained qualitatively similar results (data not shown due to space limitation). Additionally, we compared the number of proteins in overlapping clusters, i.e., clusters that have common protein members, for CASCADE was 66 (2.6%), for the maximal clique and quasi clique methods, the corresponding values were higher at 125 (5.0%) and 182 (7.2%), respectively; the other methods were not included in the comparison because they produce only non-overlapping clusters. CASCADE preformed better than the two best competing approaches, the STM and Quasi clique methods, on the Gene Ontology category as well.

These two yeast PPI datasets are relatively modular and the bottom-up approaches (e.g., Maximal clique, Quasi clique, and Rives methods) generally outperformed the top-down approaches (exemplified by the Minimum cut, Betweenness cut, and Chen methods) on functional enrichment as assessed by -*log p*. However because bottom-up approaches are based on connectivity to dense regions, the percentages of discarded nodes for the bottom-up methods are also higher than CASCADE and the top-down approaches.

The CASCADE results for the yeast DDR network [28], Rapamycin network and Rich medium network data sets [29] are also compared to the competing approaches in Table 3. We performed analysis on the functional data using the functional annotation that were acquired manually from the primary literature. The comparisons were performed on the clusters with five or more molecular components for the DNA damage response network. For the Rapamycin gene modules and Rich medium gene modules networks, the analysis was performed on the clusters with three or more molecular components because the majority of the competing methods did not yield any cluster with 5 or more members. The maximal clique method does not yield any clusters with 5 or more molecular components for the yeast DDR data set and does not yield any clusters with 3 or more molecular components for the Rapamycin network and Rich medium network data sets. For the yeast DDR network, the performance of CASCADE is comparable to Betweenness cut and Chen method, the best performing alternatives. The MCL method has comparable -*log p *values and slightly larger clusters size than the betweenness cut method but these are achieved at the cost of a high discard percentage. CASCADE also shows on average a 100-fold improved performance over the STM approach on *p*-values on biological function on these three datasets. The percentage of discarded nodes for CASCADE is 5.0%, which is significantly lower than the Quasi clique, Samanta and Liang [22] and MCL [19] methods. The percentages of nodes discarded by the Betweenness cut and minimum cut method are comparable to CASCADE. The Chen method shows the best performance on -*log p *and the lowest discard rate on the yeast DDR dataset. However, its performance appears to be sensitive to the dataset characteristics since it did not perform as well on other datasets. The yeast DDR dataset is relatively sparse and less modular than the yeast PPI network and for this reason, the top-down approaches such as Betweenness cut and minimum cut approaches have superior performance compared to the bottom-up approaches.

The Rapamycin gene modules network and the Rich medium gene modules network have low network density and clustering coefficients, and these extreme topological properties make module identification difficult. Although the Quasi clique method had the performance comparable to CASCADE on both networks, the density or merge threshold had to be set to unreasonably low values (≤ 0.4) to obtain the best clustering outcome. Because these networks are relatively small in size and have very sparse connectivity, the top-down approaches such as Betweenness cut perform relatively better.

CASCADE is a significant enhancement to STM and these two methods outperformed all the other methods on each of the datasets. Of the remaining 9 methods, the quasi clique method showed the best overall performance but its results on the sparse, less modular yeast DDR data set were poor. Thus, CASCADE is also versatile because it is robust to variations in the network topological properties such as density, clustering coefficient and size.

### Robustness Analysis

To assess robustness, the performance of CASCADE was evaluated upon addition of random interactions to unconnected protein pairs in the DIP PPI data set. Table 4 summarizes the number of clusters detected by CASCADE and the corresponding average -log *p *values for the MIPS categories. The performance of CASCADE was found to be robust to the addition of random interactions. A small decrease in the number of clusters occurred which can be attribute to the increased network connectivity upon addition of edges.

**Table 4:** Robustness Analysis.

Noise	Clusters	MIPS Function (-log*p*)	MIPS Location (-log *p*)	MIPS Complex (-log *p*)
0%	50	14.5	8.17	16.5
1%	51	13.8	7.54	15.6
2%	50	14.2	7.66	16.0
3%	49	14.4	7.71	16.7
4%	48	14.3	7.71	16.9
5%	46	14.1	7.67	16.0
10%	42	14.8	8.14	17.5

Noise column represents the percentile of random noise added into DIP PPI dataset. Clusters shows the number of clusters detected. The average -log *p *values of all detected clusters for MIPS functional, localization, and complex categories are shown.

### Computational Complexity Analysis

A comparison of the time complexity of the various methods is summarized in Table 5. The total time complexity of CASCADE is bounded by the time for QAP calculations between all pairs of nodes, which is *O*(*V *^3^*logV *+ *V *^2^*E*). In almost all biological networks, including protein-protein interaction networks, *E *= *O*(*V logV*) which makes the total complexity of CASCADE *O*(*V *^3^*logV*). Among the competing approaches, the SPC method has the best running time complexity, *O*(*V *^2^), and the minimum cut method has the worst complexity, *O*(*V *^2^*logV *+ *V E*). CASCADE uses the QAP algorithm that approximates the solution to the all possible path problem, which is *N P *hard. From this standpoint, therefore, CASCADE has good and manageable running time complexity despite being about *V *times slower than 7 of the other competing approaches: the quasi clique and maximal clique are *N P *hard. All the experiments in this paper were executed on 4 dual-core operon 2.8GHZ Linux machine. The experiments on three relatively small size data sets (Yeast DDR network, Rapamycin network, and Richmedium network) were finished in few minutes. Running time for the DIP Yeast interaction data set was 2.5 hours, and 14.3 hours for the BIOGRID yeast interaction data set.

**Table 5:** Comparison of computational complexity of CASCADE to competing clustering methods.

Method	Complexity
**CASCADE**	*O*(*V *^3^*log*(*V*))
STM	*O*(*V *^2^*log*(*V*))
Maximal clique	NP
Quasi clique	NP
Samanta	*O*(*V *^2^*log*(*V*))
Minimum cut	*O*(*V *^2^*log*(*V*) + *V E*)
Betweenness cut	*O*(*V *^2 ^+ *V E*)
MCL	*O*(*V *^2^*log*(*V*))
Chen	*O*(*V *^2 ^+ *V E*)
Rives	*O*(*V *^2^*log*(*V*))
SPC	*O*(*V *^2^)

## Discussion

In this paper, we have described and critically evaluated CASCADE, a novel clustering model for detecting functional modules from biological interaction data. In head-to-head comparisons, the CASCADE method outperforms competing approaches and is capable of effectively detecting both dense and sparsely connected, biologically relevant functional modules with fewer discards.

The existing algorithms have suffered in their clustering performance in part because they emphasize network regions of high intra-connectivity and low inter-connectivity. However, biological functional modules are not as densely connected as required for optimal performance of these methods: in the yeast PPI network, only an average of 8.7% of all potential connections between protein pairs are present within a 3rd or more specific function in MIPS functional hierarchy. The subgraphs of MIPS functional categories thus have low density and contain many singletons; some members in functional categories do not have direct physical interaction with other members of the same functional category. Thus, relative over weighting for densely connected regions can be undesirable for effective functional module detection in biological interaction data sets.

Moreover, in the PPI network, the subgraphs of actual MIPS functional categories are generally not closely congregated and tend to have longish shapes. The average diameter (which is the length of the longest path among all pairs of shortest paths) of the subgraphs of all MIPS functional categories is approximately 4 interactions long and is comparable to the average shortest paths length of 5.47 for the whole PPI network. A relative excess of emphasis on density and inter-connectivity in the existing methods can be preferential for detecting clusters with relatively balanced round shapes and limit performance. The incompleteness of clustering is another distinct drawback of existing algorithms, which produce many clusters with small size and singletons. The preference for strongly connected nodes results in many weakly connected nodes being discarded.

We examined the frequencies of individuals in each of the clusters from CASCADE (see Additional file 2 and 3). In the initial qualitative assessment in Additional file 2, the larger clusters appeared to be functionally more heterogeneous than the smaller clusters. For example, 7 of the 10 largest clusters contained "mRNA synthesis" and 6 of the 10 clusters contained "Fungal eukaryotic cell type differentiation" are constituent terms. However, there was also substantial functional cohesiveness in each large clusters, e.g., in Cluster 2, which had 303 genes, there were terms related to "DNA synthesis and replication", "Mitotic cell cycle and cell cycle control", "Modification by phosphorylation, dephosphorylation", "Phosphate utilization", "Fungal and eukaryotic cell differentiation" that evidently are related. However, the more systematic and detailed analysis in Additional file 3 did not support the premise that the larger clusters were functionally more heterogeneous than smaller clusters – the proportion of genes in the 3rd and higher levels of the MIPS hierarchy for the larger clusters was similar and unrelated to cluster size. Biologically, the "mRNA synthesis" and "Fungal eukaryotic cell type differentiation" terms have broad and pleiotropic effects and it is unsurprising that they would be required for multiple functional modules. This may better account for why CASCADE implicated them in several clusters.

## Conclusion

In conclusion, the novel occurrence probability quantification function-based metric in CASCADE accounts for both node degree and connectivity patterns and the results indicate that it is an effective approach for analyzing biological interactions.

## Methods

### Network Model

The molecular components and the biological interactions in a biological interaction data set are, respectively, represented by nodes and edges of a graph.

#### Graph definitions

An undirected graph *G *= (*V*, *E*) consists of a set *V *of nodes and a set *E *of edges, *E *⊆ *V *× *V*. An edge *e *= (*i*, *j*) connects two nodes *i *and *j*, *e *∈ *E*. The neighbors *N*(*i*) of node *i *are defined to be the set of directly connected nodes to node *i*. The degree *d*(*i*) of a node *i *is the number of the nodes connected to node *i*, |*N*(*i*)|. A path is defined as a sequence of nodes (*n*_1_..., *n*_*k*_) such that from each of its nodes there is an edge to the successor node. The length of a path is the number of edges in its node sequence. A shortest path between two nodes, *i *and *j*, is a minimal length path between them. The distance between two nodes, *i *and *j*, is the length of its shortest path.

##### The Occurrence Probability Model

We identified the Erlang distribution as a parsimonious model for describing PPI networks and other biological interactions [23,30]. A key consideration was the observation that sequentially ordered actions of protein-protein and other biological interactions are frequently observed in several biological processes. In queueing theory, the distribution of time to complete a sequence of tasks in a system with Poisson input is described by the Erlang distribution.

The occurrence probability of a sequence of pairwise interactions in the network was modeled using the Erlang distribution and queueing theory, a special case of the Gamma distribution, as follows:

(1) $$F(c)=1-e^{-\frac{x}{b}}\sum_{k=0}^{c-1}\frac{{\left(\frac{x}{b}\right)}^{k}}{k!}$$

Where *c *> 0 is the number of edges, i.e., the length of the path, between source node and the target node, *b *> 0 is the scale parameter, *x *≥ 0, is the independent variable, usually time. The occurrence probability with *x*/*b *= 1 is used. The scale parameter *b *represents the characteristic time scale required for the occurrence of an interaction between a protein pair. Thus, setting the value of *x*/*b *to unity assesses the probability that a series of interactions between a source and a target protein will occur over this characteristic time scale.

The occurrence probability function is further weighted to reflect network topology. The occurrence probability propagated by the source node is assumed to be proportional to its degree and to follow all possible paths identified using the Quasi All Paths (QAP) algorithm, which is described in the next paragraph, to the target node.

##### Quasi All Paths Enumeration Algorithm

From a biological perspective, propagating the interaction signal through all possible paths between a protein pair could be considered a more comprehensive approach for evaluating PPI networks. The Quasi All Paths (QAP) enumeration algorithm in CASCADE approximates the all possible paths problem between the node pairs in a network, and can be solved in polynomial time. The QAP enumeration algorithm, described in Procedure 1 (see Appendix), consists of iterative identification of shortest paths between a node pair. The edges located on the previously identified shortest paths are removed and the QAP procedure is repeated until the node pair is disconnected. When there is more than one shortest path between a node pair in a network, QAP selects the least resistant path based on ∏_*i *∈ *P*(*v*, *w*) _*d*(*i*) in Equation 2.

The occurrence probability function decreases rapidly as the number of edges between the source and target nodes: its values at *c *= 3 and *c *= 4 are approximately 13% and 3% of its value at *c *= 1, respectively. This suggests that it would be sufficient to compute the occurrence probability based on the first 4 terms or less in length. However, we implemented an exact implementation of the Erlang distribution because the saving in computational effort were typically minor and because Topology-Weighted Probability term required additional, stronger corrections for the degree of downstream nodes anyway.

##### The Topology-Weighted Occurrence Probability Model

During propagation to the target node through a path, the occurrence probability is assumed to dissipate at each intermediate node visited in proportion to the reciprocal of the degree on the path. The overall Topology-Weighted occurrence probability from node *v *to node *w *is defined as:

(2) $$S(v\to w)=\sum_{\rho \in QAP(v,w)}\frac{d(v)}{\prod_{i\in \rho }d(i)}F(c)$$

In Equation 2, *d*(*i*) is the degree of node *i*, *QAP*(*v*, *w*) is the set of paths identified by QAP between source node *v *and target node *w*, *ρ *is the set of the all nodes visited on a path in the *QAP*(*v*, *w*) from node *v *to node *w*, excluding the source node *v *but including target destination node *w*, and *F*(*c*) is the occurrence probability function (Equation 1).

### The CASCADE Algorithm

The pseudocode for the CASCADE algorithm, which employs the influence quantification function of Equation 2 is shown in Algorithm CASCADE. The algorithm involves four sequential processes:

#### Process 1

Compute the Topology-Weighted occurrence probability between all node pairs.

#### Process 2

Select cluster representatives for each node.

#### Process 3

Formation of preliminary clusters.

#### Process 4

Merge preliminary clusters.

Process 1 propagates the Topology-Weighted occurrence probability through Quasi All Paths, described in Procedure 1 (see Appendix), from each source node and accumulates the Topology-Weighted occurrence probability quantities on each target node for all node pairs according to Equation 2. The implementation of Process 1 is shown on lines 7–14 of the CASCADE algorithm in Algorithm CASCADE (see Appendix).

After computations of the Topology-Weighted occurrence probability propagated for all node pairs in Process 1, each node selects the nodes with the highest occurrence probability quantity as its representative to the cluster in Process 2. Preliminary clusters are generated in Process 3 by accumulating each node toward its representative. Lines from 15–24 in Algorithm CASCADE contain the representative selection process and the preliminary cluster formation process.

Process 4, summarized in the Merge process in Procedure 2 (see Appendix), iteratively merges preliminary cluster pairs with significant interconnections and overlaps. The findMaxPair method finds the pair with most interconnections between them. The Merge process then merges the pair and updates the cluster list. The Merge process continues until the interconnections and overlaps of all cluster pairs satisfy the predefined threshold.

In the final Merge process described in Procedure 2, CASCADE takes inter-connectivity among detected preliminary clusters into consideration to find topologically more refined clusters. As illustrated in Figure 2, CASCADE counts the edges inter-connecting between a preliminary cluster pair. According to our definition of inter-connection edges between two clusters in Figure 2, we consider various types of inter-connecting edges, i.e., not only the edges between mutually exclusive nodes but also the edges among overlapping nodes and mutually exclusive nodes etc. The degree of inter-connectivity between clusters by the similarity of two clusters *C*_*i *_and *C*_*j *_defined as:

(3) $$Similarity(C_{i},C_{j})=\frac{interconnectivity(C_{i},C_{j})}{minisize(C_{i},C_{j})}$$

where *interconnectivity*(*C*_*i*_, *C*_*j*_) is the number of edges between clusters *C*_*i *_and *C*_*j*_, and *minsize*(*C*_*i*_, *C*_*j*_) is the size of the smaller cluster among clusters *C*_*i*_and *C*_*j*_. The *Similarity*(*C*_*i*_, *C*_*j*_) between two clusters *C*_*i *_and *C*_*j *_is the ratio of the number of the edges between them to the size of the smaller cluster. Highly interconnected clusters are iteratively merged based on the similarity of the clusters. The pair of clusters that have the highest similarity are merged in each iteration and the merge process iterates until the highest similarity of all cluster pairs is less than a given threshold. The cluster pair with the biggest difference in cluster size was first merged if there are more than one cluster pair that have the same similarity values.

### Cluster Assessment

The structures of the clusters identified by CASCADE and other competing alternative approaches are assessed using several metrics. The clustering coefficient, *C*(*v*), of a node *v *measures the connectivity among its direct neighbors:

(4) $$C(v)=\frac{2\left|\cup_{i,j\in N(v)}(i,j)\right|}{d(v)(d(v)-1)}$$

In Equation 4, *N*(*v*) is the set of the direct neighbors of node *v *and *d*(*v*) is the number of the direct neighbors of node *v*. Highly connected nodes have high values of clustering coefficient.

The betweenness centrality, *C*_*B*_(*v*), is a measure of the global importance of a node that assesses the proportion of shortest paths between all node pairs that pass through the node of interest [31]. The betweenness centrality, *C*_*B*_(*v*) for a node of interest, *v*, is defined by:

(5) $$C_{B}(v)=\sum_{s\neq v\neq t\in V}\frac{\rho_{st}(v)}{\rho_{st}}$$

In Equation 5, *ρ*_*st *_is the number of shortest paths from node *s *to *t *and *ρ*_*st*_(*v*) the number of shortest paths from *s *to *t *that pass through the node *v*.

The extent to which the clusters are associated with a specific biological function is evaluated using a *p*-value based on the hypergeometric distribution [15]. The *p*-value is the probability that a cluster would be enriched with proteins with a particular function by chance alone. The *p*-value is given by

(6) $$p=1-\sum_{i=0}^{k-1}\frac{\left(\begin{array}{c} C\\ i \end{array}\right)\left(\begin{array}{c} G-C\\ n-i \end{array}\right)}{\left(\begin{array}{c} G\\ n \end{array}\right)}$$

In Equation 6, *C *is the size of the cluster containing *k *proteins with a given function; *G *is the size of the universal set of proteins of known proteins and contains *n *proteins with the function. In this paper, all *p*-values were corrected for multiple hypothesis testing, Benjamini Hochberg method [32]. Because the *p*-values are frequently small numbers with positive values between 0 and 1, the negative logarithms (to base 10, denoted -*log p*) are used. A -*log p *value of 2 or greater indicates statistical significance at *α *= 0.01.

The density of subgraphs of functional categories is measured by:

(7) $$D_{s}=\frac{2e}{n(n-1)}$$

In Equation 7, *n *is the number of nodes and *e *is the number of interactions in a subgraph *s *of a biological network.

The lethality data for the yeast PPI data set are obtained from MIPS database, which lists whether yeast strains that are deficient for specific proteins are viable or not.

### Programming and Code

The coding and running for CASCADE and the other clustering methods except MCL and SPC were conducted in the Java programming language on the Linux operating system. The source code for MCL was obtained from *micans *[33]. The SPC source code was obtained from Virtual Computational Chemistry Laboratory [34] and was conducted on the Solaris system.

### Biological Interaction Data

The DIP core yeast (*S. cerevisiae*) PPI data set was obtained from the DIP database [24]. This dataset includes 2526 proteins and 5949 filtered reliable physical interactions. The Biogrid yeast PPI dataset, which has 5390 proteins and 56860 interactions, was obtained from BioGrid [27]. Three other smaller but experimentally well-characterized PPI data sets were also assessed. The yeast DNA damage response (DDR) network (96 nodes, 133 edges) and the corresponding function categories were manually extracted by inspection of Figure 5 in [28]. The Rich medium gene modules network (111 nodes, 147 edges), Rapamycin gene modules network (50 nodes, 88 edges) and their corresponding functional categories were manually extracted by inspection of Figures 1 and 4 in [29]. MIPS

categories (03/16/2006 version) were obtained from MIPS public database [21]. Gene Ontology data were obtained from the Gene Ontology database [35].

## Authors' contributions

WCH designed the algorithm, performed the experiments and the analysis, and drafted the manuscript. YRC partly designed the algorithm and analyzed the results. MR partly designed the algorithm and analyzed the results. MR and AZ coordinated the project and revised the final manuscript. All authors read and approved the final manuscript.

## Appendix

### Algorithm 1. CASCADE(G)

1: V: set of nodes in graph G

2: *F*(*c*): The occurrence probability function

3: *S*(*v *→ *w*): The occurrence probability arrived from source protein *v *to target protein *w*

4: QAP(*v*, *w*): list of paths between protein *v *and *w *identified by QAP algorithm

5: Clusters: the list of final clusters

6: PreClusters: the list of preliminary clusters

7: **for ***each node pair*(*v*, *w*) *v*, *w *∈ *V*, *v *≠ *w ***do**

8:    QAP(*v*, *w*) = QAP(G, *v*, *w*)

9:    $S(v\to w)=\sum_{\rho \in QAP(v,w)}\frac{d(v)}{\prod_{i\in \rho }d(i)}F(c)$

10: **end for**

11: **for ***each node v *∈ *V ***do**

12:    *v.representative *⇐ select the best scored node *w *for node *v*

13:    **if ***cluster_w *== *null ***then**

14:       Make *cluster w*

15:       *cluster_w.add*(*v*)

16:       *PreClusters.add*(*cluster_w*)

17:    **else**

18:       *cluster_w.add*(*v*)

19:    **end if**

20: **end for**

21: Clusters ⇐ **Merge**(PreClusters)

### Procedure 1. QAP(G, s, t)

1: G: a graph

2: *s*: source node

3: *t*: target node

4: shortest_path(*s*, *t*): a shortest path between a node pair *s *and *t *in graph G

5: edge_list: list of edges

6: QAPs: list of paths

7: **while ***node s and node t is disconnected ***do**

8:    Find shortest_path(*s*, *t*)

9:    Add shortest_path(*s*, *t*) to QAPs

10:    Add all edges on shortest_path(*s*, *t*) to edge_list

11:    Remove all edges on shortest_path(*s*, *t*) from graph G

12: **end while**

13: Restore all edges in edge_list into graph G

14: **return **QAPs

### Procedure 2. Merge(Clusters)

1: Clusters: the cluster list

2: MaxPair: the cluster pair(*m*, *n*) with max interconnections among all pairs

3: Max.value: interconnections between cluster pair *m *and *n*

4: MaxPair ⇐ findMaxPair(Clusters, null)

5: **while ***Max.value *≥ *threshold ***do**

6:    NewCluster ⇐ merge MaxPair *m *and *n*

7:    Replace cluster *m *with NewCluster

8:    Remove cluster *n*

9:    MaxPair ⇐ findMaxPair(Clusters, NewCluster)

10: **end while**

11: **return **Clusters

## Supplementary Material

Additional file 1 Clusters obtained using CASCADE for the yeast PPI network. The first column is a cluster identifier; the Size column indicates the number of proteins in each cluster; the Density indicates the percentage of possible protein interactions that are present; the *H *column indicates the percentage of proteins concordant with the major function indicated in the last column; the *D *column indicates the percentage of proteins discordant with the major function and *U *column indicates the percentage of proteins not assigned to any function. The -log *p *values for biological function are shown. Click here for file

Additional file 2 Functional term distribution. Functional term distribution in MIPS functional category for the top 10 largest clusters in Additional File 1. (a) cluster 1, size 411. (b) cluster 2, size 303. (c) cluster 3, size 240. (d) cluster 4, size 176. (e) cluster 5, size 170. (f) cluster 6, size 104. (g) cluster 7, size 96. (h) cluster 8, size 79. (i) cluster 9, size 78. (j) cluster 10, size 73. Each figure presents the percentile of proteins that are accordant with the top ten best accordant functional terms for each cluster. Click here for file

Additional file 3 Normalized number of functional terms for each cluster detected by CACASDE. The first column is a cluster identifier; the Size column indicates the number of proteins in each cluster. The normalized numbers of functional terms in the MIPS functional hierarchy for each identified cluster are presented in the third, the fourth, and the fifth column. The number of functional terms per each cluster is normalized by its cluster size. The third column represents the normalized number of functional terms that are more specific than 2nd level functional hierarchy. The fourth column represents the normalized number of functional terms that are more specific than 3rd level functional hierarchy. The fifth column represents the normalized number of functional terms that are more specific than 4th level functional hierarchy. Click here for file

Additional file 4 Topological shape of a cluster and its functional annotations. Cluster 20 in Additional File 1. (a) sub graph of Cluster 20 extracted from DIP PPI network. Each protein is annotated by MIPS functional category. (b) MIPS functional IDs and their corresponding literal names. The best accordant functional term is boldfaced. Click here for file

Additional file 5 Topological shape of a cluster and its functional annotations. Cluster 21 in Additional File 1. (a) sub graph of Cluster 21 extracted from DIP PPI network. Each protein is annotated by MIPS functional category. (b) MIPS functional IDs and their corresponding literal names. The best accordant functional term is boldfaced. Click here for file

Additional file 6 Topological shape of a cluster and its functional annotations. Cluster 22 in Additional File 1. (a) sub graph of Cluster 22 extracted from DIP PPI network. Each protein is annotated by MIPS functional category. (b) MIPS functional IDs and their corresponding literal names. The best accordant functional term is boldfaced. Click here for file

Additional file 7 Topological shape of a cluster and its functional annotations. Cluster 25 in Additional File 1. (a) sub graph of Cluster 25 extracted from DIP PPI network. Each protein is annotated by MIPS functional category. (b) MIPS functional IDs and their corresponding literal names. The best accordant functional term is boldfaced. Click here for file

## References

[B1] Gavin AC (2002). Functional organization of the yeast proteome by systematic analysis of protein complexes. Nature.

[B2] Ho Y (2002). Systematic identification of protein complexes in Saccharomyces cerevisiae by mass spectrometry. Nature.

[B3] Ito T (2001). A comprehensive two-hybrid analysis to explore the yeast protein interactome. PNAS.

[B4] Uetz P (2000). A comprehensive analysis of protein-protein interactions in Saccharomyces cerevisiae. Nature.

[B5] Gavin AC (2006). Proteome survey reveals modularity of the yeastcell machinery. Nature.

[B6] Hartwell LH, Hopfield JJ, Leibler S, Murray AW (1999). From molecular to modular cell biology. Nature.

[B7] Snel B, Bork P, Huynen MA (2002). The identification of functional modules from the genomic association of genes. PNAS.

[B8] Rives AW, Galitski T (2003). Modular organization of cellular networks. Proc Natl Acad Sci.

[B9] Zhou H (2003). Distance, dissimilarity index, and network community structure. Phys Rev E Stat Nonlin Soft Matter Phys.

[B10] Wu H, Su Z, Mao F, Olman V, Xu Y (2005). Prediction of functional modules based on comparative genome analysis and Gene Ontology application. Nucleic Acids Res.

[B11] Blatt M, Wiseman S, Domany E (1996). Superparamagnetic Clustering of Data. Phys Rev Lett.

[B12] Tornow S, Mewes HW (2003). Functional modules by relating protein interaction networks and gene expression. Nucleic Acids Res.

[B13] Chen J, Yuan B (2006). Detecting functional modules in the yeast protein-protein interaction network. Bioinformatics.

[B14] Spirin V, Mirny LA (2003). Protein complexes and functional modules in molecular networks. PNAS.

[B15] Bu D, Zhao Y, Cai L, Xue H, Zhu X, Lu H, Zhang J, Sun S, Ling L, Zhang N, Li G, Chen R (2003). Topological structure analysis of the protein-protein interaction network in budding yeast. Nucleic Acids Res.

[B16] Bader GD, Hogue CW (2003). An automated method for finding molecular complexes in large protein interaction networks. BMC Bioinformatics.

[B17] King AD, Przulj N, Jurisica I (2004). Protein complex prediction via cost-based clustering. Bioinformatics.

[B18] Hartuv E, Shamir R (2000). A Clustering Algorithm based Graph Connectivity. Information Processing Letters.

[B19] van Dongen S (2000). Technical Report INS-R0010: A cluster algorithm for graphs.

[B20] Pereira-Leal JB, Enright AJ, Ouzounis CA (2004). Detection of functional modules from protein interaction networks. Proteins.

[B21] Mewes HW, Frishman D, Mayer KF, Munsterkotter M, Noubibou O, Pagel P, Rattei T, Oesterheld M, Ruepp A, Stumpflen V (2006). MIPS: analysis and annotation of proteins from whole genome in 2005. Nucleic Acid Research.

[B22] Samanta MP, Liang S (2003). Redundancies in large-scale protein interaction networks. Proc Natl Acad Sci.

[B23] Hwang W, Cho YR, Zhang A, Ramanathan M (2006). A novel functional module detection algorithm for protein-protein interaction networks. Algorithms Mol Biol.

[B24] Deane CM, Salwinski L, Xenarios I, Eisenberg D (2002). Protein interactions: two methods for assessment of the reliability of high throughput observations. Mol Cell Proteomics.

[B25] Johnson DB (1977). Efficient algorithms for shortest paths in sparse networks. J of the ACM.

[B26] Girvan M, Newman MEJ (2002). Community structure in social and biological networks. PNAS.

[B27] Stark C, Breitkreutz B, Reguly T, Boucher L, Breitkreutz A, Tyers M (2003). BioGRID: A General Repository for Interaction Datasets. Nucleic Acids Res.

[B28] Workman CT, Mak HC, McCuine S, Tagne JB, Agarwal M, Ozier O, Begley TJ, Samson LD, Ideker T (2006). A systems approach to mapping DNA damage response pathways. Science.

[B29] Bar-Joseph Z, Gerber GK, Lee TI, Rinaldi NJ, Yoo JY, Robert F, Gordon DB, Fraenkel E, Jaakkola TS, Young RA, Gifford DK (2003). Computational discovery of gene modules and regulatory networks. Nature Biotechnology.

[B30] Johnson NL, Kotz S, Balakrishnan N (1994). Continuous univariate distributions.

[B31] Freeman LC (1979). A set of measures of centrality based on betweenness. Sociometry.

[B32] Benjamini Y, Hochberg Y (1995). Controlling the false discovery rate: a practical and powerful approach to multiple testing. Journal of the Royal Statistical Society.

[B33] micans. http://micans.org/mcl/.

[B34] Virtual Computational Chemistry Laboratory. http://www.vcclab.org/.

[B35] The Gene Ontology Consortium (2000). Gene Ontology: tool for the unification of biology. Nature Genet.

