# Supplementary material for: CASCADE: a novel quasi all paths-based network analysis algorithm for clustering biological interactions
Source: BMC Bioinformatics. 2008 Jan 29;9:64. doi: 10.1186/1471-2105-9-64 (PMC2253513; doi:10.1186/1471-2105-9-64)
Supplement: Additional file 1 — Clusters obtained using CASCADE for the yeast PPI network. The first column is a cluster identifier; the Size column indicates the number of proteins in each cluster; the Density indicates the percentage of possible protein interactions that are present; the H column indicates the percentage of proteins concordant with the major function indicated in the last column; the D column indicates the percentage of proteins discordant with the major function and U column indicates the percentage of proteins not assigned to any function. The -log p values for biological function are shown. [file 1471-2105-9-64-S1.pdf]

Cluster	Size	Density	Distribution H	D	U	-Log$p$	Function
1	411	0.0103	17.5	76.4	6.1	19.3	Vesicular transport
2	303	0.0104	33.3	60.0	6.6	19.9	Mitotic cell cycle & cell cycle control
3	240	0.0171	23.3	70.8	5.8	44.1	Nuclear transport
4	176	0.0274	46.0	43.1	10.8	30.8	Transported compounds
5	170	0.0181	32.4	60.0	7.6	19.0	Cytoskeleton
6	104	0.0220	14.8	76.5	8.7	16.3	Conversion to kinetic energy
7	96	0.0450	76.0	19.8	4.2	39.7	mRNA synthesis
8	79	0.0431	58.2	39.2	2.5	33.3	General transcription activities
9	78	0.0416	35.9	62.8	1.3	19.9	Ribosome biogenesis
10	73	0.0353	39.7	58.9	1.5	9.7	Phosphate metabolism
11	70	0.0356	22.9	65.7	11.4	8.1	Ribosome biogenesis
12	69	0.0682	66.7	24.6	8.7	43.9	mRNA processing (splicing, 5'-, 3'-end processing)
13	60	0.0616	23.3	65.0	11.7	13.7	Homeostasis of protons
14	50	0.0637	68.0	30.0	2.0	34.0	rRNA processing
15	37	0.0781	10.8	89.2	0.0	7.2	Cell-cell adhesion
16	29	0.1330	48.3	51.7	0.0	26.8	Peroxisomal transport
17	28	0.1164	28.6	67.9	3.6	6.9	Cytokinesis (cell division) /septum formation
18	23	0.1581	65.2	30.4	4.3	13.6	DNA conformation modification (e.g. chromatin)
19	18	0.1764	72.2	22.2	5.6	18.2	Mitochondrial transport
20	17	0.2206	70.6	29.4	0.0	22.5	Microtubule cytoskeleton
21	17	0.2206	82.4	11.8	5.9	19.1	rRNA synthesis
22	16	0.3000	93.8	6.2	0.0	19.5	Splicing
23	15	0.2190	26.7	73.3	0.0	30.4	Regulation of nitrogen utilization
24	15	0.3047	86.7	13.3	0.0	8.1	Energy generation (e.g. ATP synthase)
25	14	0.3407	85.7	14.3	0.0	14.3	DNA conformation modification (e.g. chromatin)
26	14	0.1978	57.1	28.6	14.3	13.3	chromosome condensation
27	13	0.5641	76.9	23.1	0.0	17.0	Mitosis
28	13	0.4103	69.2	23.1	7.7	15.4	3'-end processing
29	12	0.3636	58.3	41.7	0.0	14.3	Posttranslational modification of amino acids
30	12	0.1667	16.7	75.0	8.3	2.3	Autoproteolytic processing
31	11	0.2181	54.5	45.4	0.0	2.9	Transcriptional control
32	10	0.4667	80.0	20.0	0.0	14.3	Translation initiation
33	9	0.2500	22.2	77.8	0.0	4.1	S-adenosyl-methionine - homocysteine cycle
34	8	0.3214	50.0	37.5	12.5	5.5	Metabolism of energy reserves (e.g. glycogen, trehalose)
35	8	0.2857	62.5	25.0	12.5	5.2	Vacuolar transport
36	7	0.3333	42.9	57.1	0.0	7.1	DNA damage response
37	7	0.3333	71.4	28.6	0.0	4.3	Modification by ubiquitination, deubiquitination
38	7	0.2857	28.6	71.4	0.0	3.4	Biosynthesis of serine
39	6	0.5333	100.0	0.0	0.0	12.1	Modification with sugar residues (e.g. glycosylation)
40	6	0.4000	100.0	0.0	0.0	10.0	ER to Golgi transport
41	6	0.3333	16.7	16.7	66.6	7.0	Regulation of nitrogen utilization
42	6	0.4667	100.0	0.0	0.0	3.9	DNA recombination and DNA repair
43	6	0.4000	66.6	33.3	0.0	1.9	Intracellular signalling
44	5	0.5000	100.0	0.0	0.0	6.8	Transported compounds (substrates)
45	5	0.4000	100.0	0.0	0.0	6.8	Meiosis
46	5	0.6000	100.0	0.0	0.0	6.0	Vacuolar transport
47	5	0.9000	100.0	0.0	0.0	4.5	Vesicular transport (Golgi network, etc.)
48	5	0.4000	20.0	40.0	40.0	4.0	cAMP mediated signal transduction
49	5	0.5000	40.0	40.0	20.0	3.1	Oxidative stress response
50	5	0.4000	40.0	60.0	0.0	1.8	Deoxyribonucleotide metabolism

Table 1. Clusters obtained using CASCADE for the yeast PPI network. The first column is a cluster identifier; the Size column indicates the number of proteins in each cluster; the Density indicates the percentage of possible protein interactions that are present; the H column indicates the percentage of proteins concordant with the major function indicated in the last column; the D column indicates the percentage of proteins discordant with the major function and U column indicates the percentage of proteins not assigned to any function. The -log p values for biological function are shown.
