# Supplementary material for: CASCADE: a novel quasi all paths-based network analysis algorithm for clustering biological interactions
Source: BMC Bioinformatics. 2008 Jan 29;9:64. doi: 10.1186/1471-2105-9-64 (PMC2253513; doi:10.1186/1471-2105-9-64)
Supplement: Additional file 2 — Functional term distribution. Functional term distribution in MIPS functional category for the top 10 largest clusters in Additional File 1. (a) cluster 1, size 411. (b) cluster 2, size 303. (c) cluster 3, size 240. (d) cluster 4, size 176. (e) cluster 5, size 170. (f) cluster 6, size 104. (g) cluster 7, size 96. (h) cluster 8, size 79. (i) cluster 9, size 78. (j) cluster 10, size 73. Each figure presents the percentile of proteins that are accordant with the top ten best accordant functional terms for each cluster. [file 1471-2105-9-64-S2.pdf]

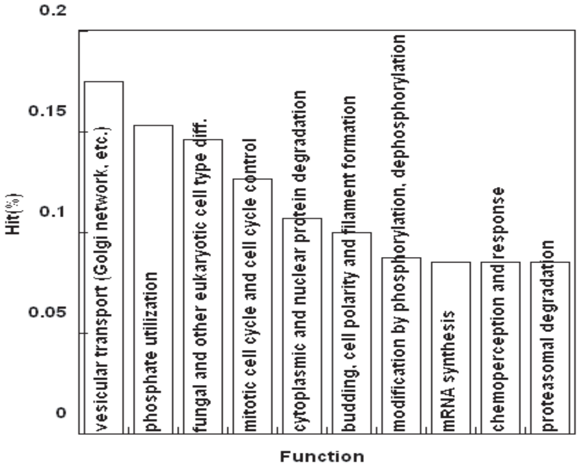
0.2
0.15
0.1
0.05
0
Hit(%)
vesicular transport (Golgi network, etc.)
phosphate utilization
fungal and other eukaryotic cell type diff.
mitotic cell cycle and cell cycle control
cytoplasmic and nuclear protein degradation
budding, cell polarity and filament formation
modification by phosphorylation, dephosphorylation
mRNA synthesis
chemoperception and response
proteasomal degradation
Function
(a)

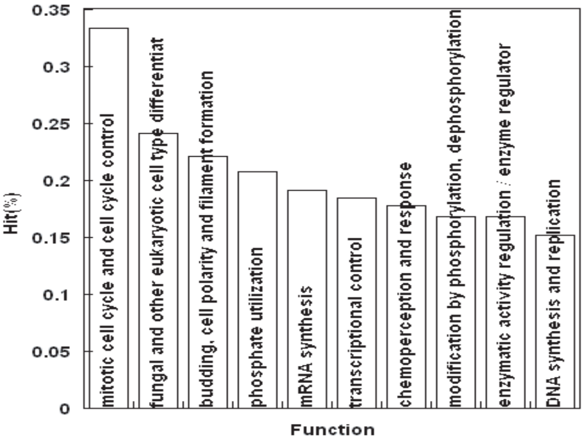
0.35
0.3
0.25
0.2
0.15
0.1
0.05
0
Hit(%)
mitotic cell cycle and cell cycle control
fungal and other eukaryotic cell type differentiat
budding, cell polarity and filament formation
phosphate utilization
mRNA synthesis
transcriptional control
chemoperception and response
modification by phosphorylation, dephosphorylation
enzymatic activity regulation / enzyme regulator
DNA synthesis and replication
Function
(b)

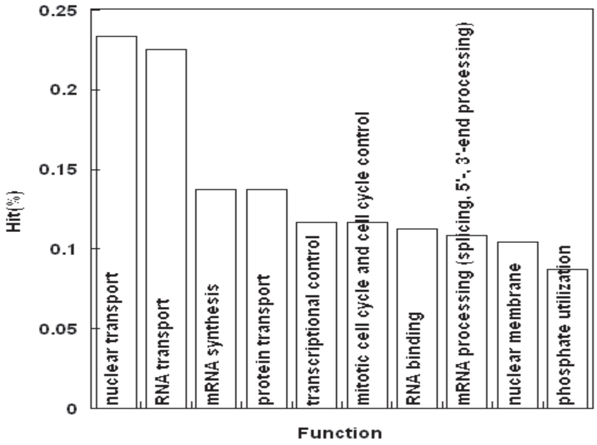
0.25
0.2
0.15
0.1
0.05
0
Hit(%)
nuclear transport
RNA transport
mRNA synthesis
protein transport
transcriptional control
mitotic cell cycle and cell cycle control
RNA binding
mRNA processing (splicing, 5'-, 3'-end processing)
nuclear membrane
phosphate utilization
Function
(c)

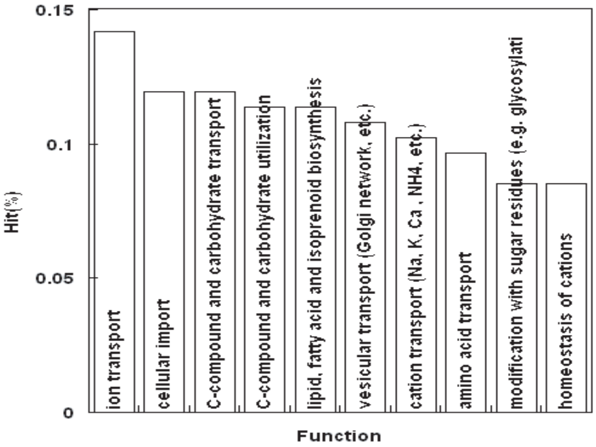
0.15
0.1
0.05
0
Hit(%)
ion transport
cellular import
C-compound and carbohydrate transport
C-compound and carbohydrate utilization
lipid, fatty acid and isoprenoid biosynthesis
vesicular transport (Golgi network, etc.)
cation transport (Na, K, Ca , NH4, etc.)
amino acid transport
modification with sugar residues (e.g. glycosylati
homeostasis of cations
Function
(d)

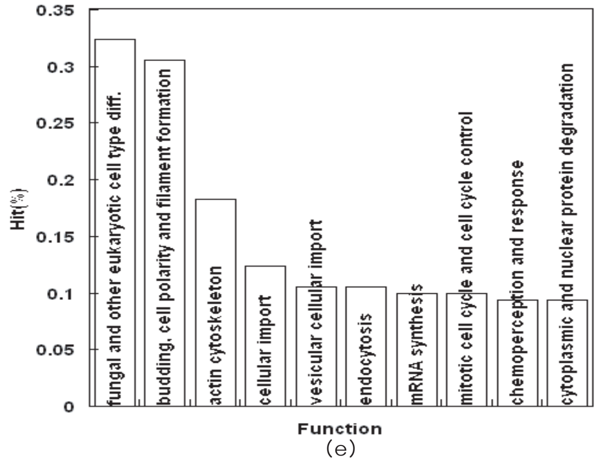
0.35
0.3
0.25
0.2
0.15
0.1
0.05
0
Hit(%)
fungal and other eukaryotic cell type diff.
budding, cell polarity and filament formation
actin cytoskeleton
cellular import
vesicular cellular import
endocytosis
mRNA synthesis
mitotic cell cycle and cell cycle control
chemoperception and response
cytoplasmic and nuclear protein degradation
Function
(e)

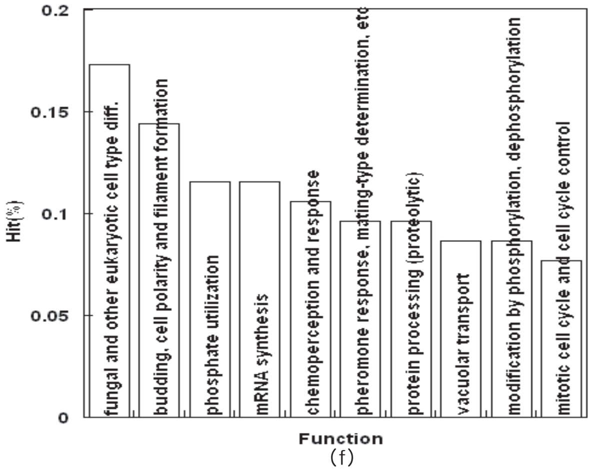
0.2
0.15
0.1
0.05
0
Hit(%)
fungal and other eukaryotic cell type diff.
budding, cell polarity and filament formation
phosphate utilization
mRNA synthesis
chemoperception and response
pheromone response, mating-type determination, etc
protein processing (proteolytic)
vacuolar transport
modification by phosphorylation, dephosphorylation
mitotic cell cycle and cell cycle control
Function
(f)

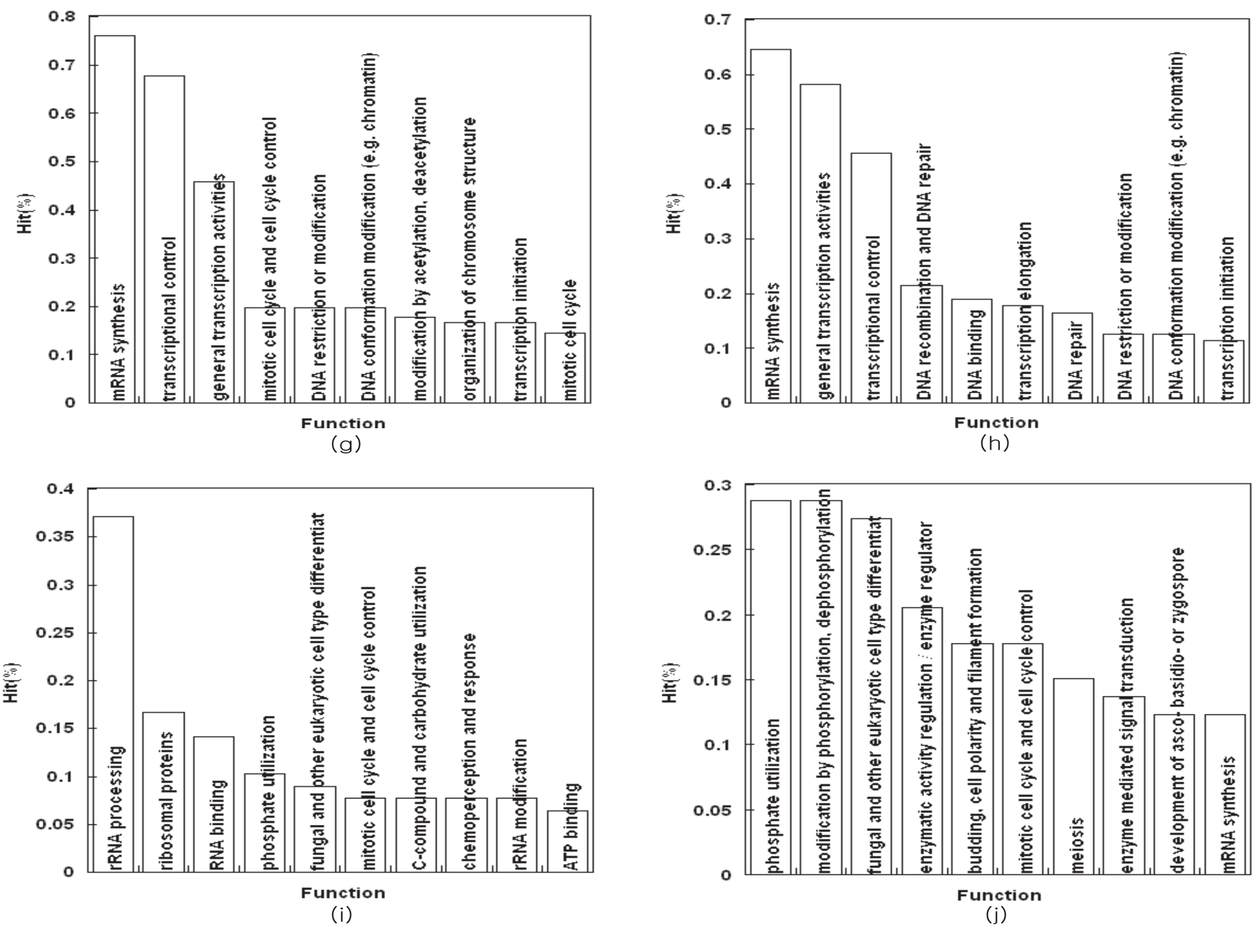

Fig. 1: Functional term distribution in MIPS functional category for the 10 largest clusters detected by CASCADE (Additional file 1). (a) cluster 1, size 411. (b) cluster 2, size 303. (c) cluster 3, size 240. (d) cluster 4, size 176. (e) cluster 5, size 170. (f) cluster 6, size 104. (g) cluster 7, size 96. (h) cluster 8, size 79. (i) cluster 9, size 78. (j) cluster 10, size 73. Each histogram shows the percentile of proteins that are annotated by the top ten best assigned functional terms for each cluster.
