# Supplementary material for: CASCADE: a novel quasi all paths-based network analysis algorithm for clustering biological interactions
Source: BMC Bioinformatics. 2008 Jan 29;9:64. doi: 10.1186/1471-2105-9-64 (PMC2253513; doi:10.1186/1471-2105-9-64)
Supplement: Additional file 3 — Normalized number of functional terms for each cluster detected by CACASDE. The first column is a cluster identifier; the Size column indicates the number of proteins in each cluster. The normalized numbers of functional terms in the MIPS functional hierarchy for each identified cluster are presented in the third, the fourth, and the fifth column. The number of functional terms per each cluster is normalized by its cluster size. The third column represents the normalized number of functional terms that are more specific than 2nd level functional hierarchy. The fourth column represents the normalized number of functional terms that are more specific than 3rd level functional hierarchy. The fifth column represents the normalized number of functional terms that are more specific than 4th level functional hierarchy. [file 1471-2105-9-64-S3.pdf]

Cluster	Size	≥ 3rd hierarchy	≥ 4th hierarchy	≥ 5th hierarchy
1	411	0.38	0.17	0.06
2	303	0.41	0.21	0.07
3	240	0.46	0.21	0.04
4	176	0.39	0.17	0.04
5	170	0.52	0.21	0.05
6	104	0.74	0.29	0.09
7	96	0.50	0.16	0.04
8	79	0.48	0.19	0.04
9	78	0.54	0.18	0.01
10	73	0.81	0.36	0.12
11	70	0.64	0.24	0.07
12	69	0.22	0.06	0.0
13	60	0.67	0.28	0.05
14	50	0.26	0.06	0.0
15	37	0.89	0.30	0.05
16	29	0.24	0.07	0.0
17	28	0.79	0.29	0.04
18	23	0.57	0.13	0.0
19	18	0.33	0.11	0.0
20	17	0.35	0.18	0.06
21	17	0.29	0.06	0.0
22	16	0.25	0.06	0.0
23	15	1.13	0.53	0.20
24	15	0.60	0.13	0.0
25	14	0.79	0.29	0.14
26	14	0.64	0.21	0.0
27	13	0.69	0.31	0.08
28	13	0.54	0.23	0.08
29	12	1.17	0.50	0.17
30	12	0.42	0.17	0.0
31	11	0.82	0.45	0.1
32	10	0.10	0.0	0.0
33	9	0.78	0.44	0.11
34	8	0.50	0.13	0.0
35	8	0.63	0.50	0.25
36	7	1.43	0.29	0.0
37	7	0.86	0.29	0.0
38	7	1.57	0.86	0.29
39	6	1.33	0.50	0.0
40	6	1.00	0.50	0.0
41	6	0.83	0.33	0.0
42	6	0.33	0.17	0.0
43	6	0.0	0.0	0.0
44	5	0.60	0.20	0.0
45	5	0.40	0.20	0.0
46	5	0.40	0.0	0.0
47	5	0.20	0.0	0.0
48	5	1.00	0.0	0.0
49	5	2.60	1.00	0.4
50	5	0.40	0.0	0.0

Table 2. Normalized number of functional terms for each cluster detected by CACASDE (Additional file 1). The first column is a cluster identifier; the Size column indicates the number of proteins in each cluster. The normalized numbers of functional terms in the MIPS functional hierarchy for each identified cluster are presented in the third, the fourth, and the fifth column. The number of functional terms per each cluster is normalized by its cluster size. The third column represents the normalized number of functional terms that are more specific than 2nd level functional hierarchy. The fourth column represents the normalized number of functional terms that are more specific than 3rd level functional hierarchy. The fifth column represents the normalized number of functional terms that are more specific than 4th level functional hierarchy.

1 SALIENT RESULTS FROM SUPPLEMENTARY TABLE 2

The mean of the normalized number of functional terms is 0.65 and the median is 0.57 for the 3rd or more specific hierarchy in the third column. The mean of the normalized number of functional terms is 0.24 and the median is 0.21 for the 4th or more specific hierarchy in the fourth column. The mean of the normalized number of functional terms is 0.06 and the median is 0.04 for the 5th or more specific hierarchy in the fifth column. The average functional terms annotated for each protein in the PPI network was also measured and showed 6.58. It means that a protein is annotated by more than 6 functional terms in MIPS functional category. This normalized number of functional terms shows the functional heterogeneity of proteins in each cluster. The functional homogeneity of the identified clusters showed good performance considering their large cluster size.
