# Supplementary material for: CASCADE: a novel quasi all paths-based network analysis algorithm for clustering biological interactions
Source: BMC Bioinformatics. 2008 Jan 29;9:64. doi: 10.1186/1471-2105-9-64 (PMC2253513; doi:10.1186/1471-2105-9-64)
Supplement: Additional file 4 — Topological shape of a cluster and its functional annotations. Cluster 20 in Additional File 1. (a) sub graph of Cluster 20 extracted from DIP PPI network. Each protein is annotated by MIPS functional category. (b) MIPS functional IDs and their corresponding literal names. The best accordant functional term is boldfaced. [file 1471-2105-9-64-S4.pdf]

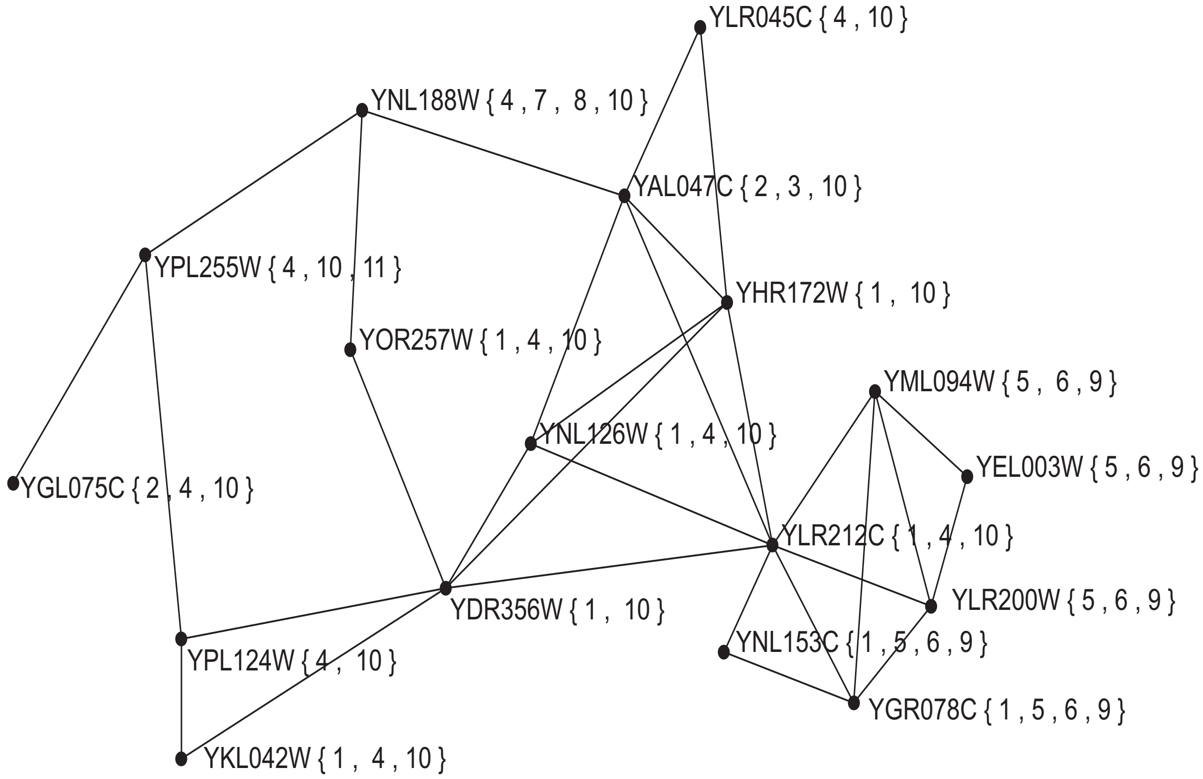

(a)

Fucntion ID	MIPS ID	Function name
1	10.03.01	mitotic cell cycle and cell cycle control
2	10.03.01.01.11	mitosis
3	10.03.04.09	nuclear migration
4	10.03.05.01	spindle pole body/centrosome and microtubule cycle
5	14.01	protein folding and stabilization
6	16.01	protein binding
7	34.11.03.07	pheromone response, mating-type determination, sex-specific proteins
8	41.01.01	mating (fertilization)
9	42.04	cytoskeleton
**10**	**42.04.05**	**microtubule cytoskeleton**
11	43.01.03.05	budding, cell polarity and filament formation

(b)

Fig. 2: Topological shape and functional annotations of Cluster 20 in Additional file 1. (a) sub graph of Cluster 20 extracted from DIP PPI network. Each protein is annotated by MIPS functional category. (b) MIPS functional IDs and their corresponding literal names. The best assigned functional term is boldfaced.
