# Supplementary material for: CASCADE: a novel quasi all paths-based network analysis algorithm for clustering biological interactions
Source: BMC Bioinformatics. 2008 Jan 29;9:64. doi: 10.1186/1471-2105-9-64 (PMC2253513; doi:10.1186/1471-2105-9-64)
Supplement: Additional file 5 — Topological shape of a cluster and its functional annotations. Cluster 21 in Additional File 1. (a) sub graph of Cluster 21 extracted from DIP PPI network. Each protein is annotated by MIPS functional category. (b) MIPS functional IDs and their corresponding literal names. The best accordant functional term is boldfaced. [file 1471-2105-9-64-S5.pdf]

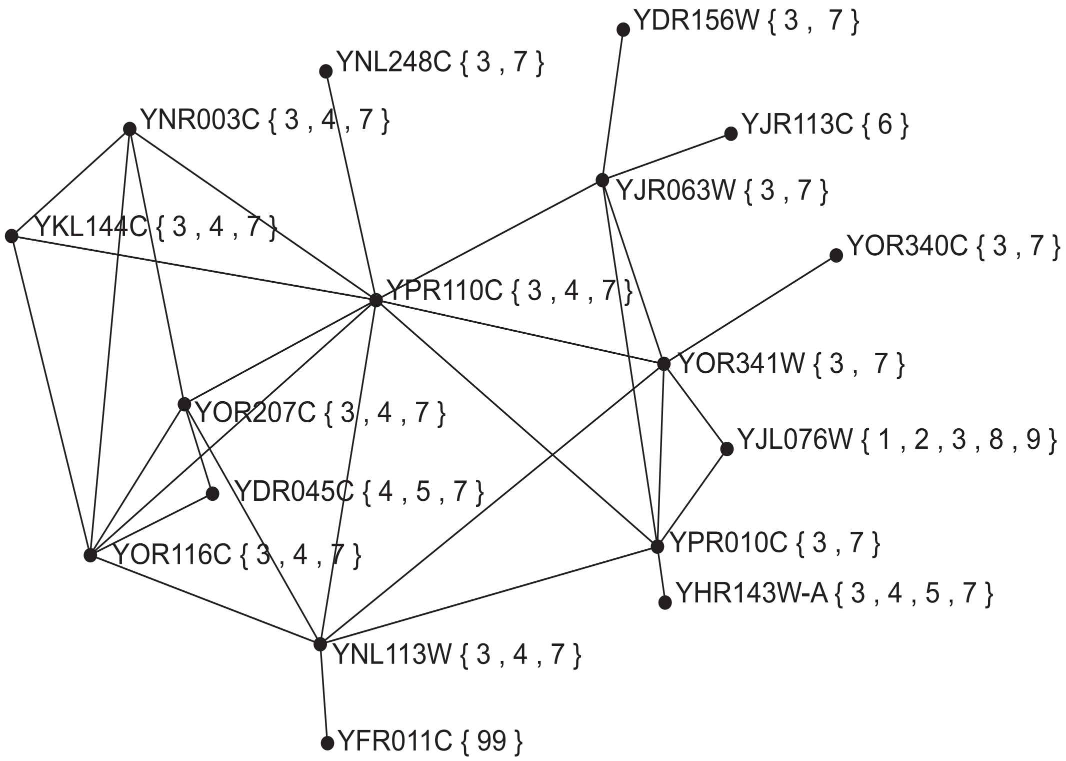

(a)

Function ID	MIPS ID	Function name
1	10.01.09.05	DNA conformation modification (e.g. chromatin)
2	10.03.01	mitotic cell cycle and cell cycle control
**3**	**11.02.01**	**rRNA synthesis**
4	11.02.02	tRNA synthesis
5	11.02.03.01	general transcription activities
6	12.01.01	ribosomal proteins
7	16.03.01	sugar binding
8	16.03.03	fatty acid binding (e.g. acyl-carrier protein)
9	42.10.07	nucleolus
99	99	Unknown

(b)

Fig. 3: Topological shape and functional annotations of Cluster 21 in Additional file 1. (a) sub graph of Cluster 21 extracted from DIP PPI network. Each protein is annotated by MIPS functional category. (b) MIPS functional IDs and their corresponding literal names. The best assigned functional term is boldfaced.
