# Supplementary material for: CASCADE: a novel quasi all paths-based network analysis algorithm for clustering biological interactions
Source: BMC Bioinformatics. 2008 Jan 29;9:64. doi: 10.1186/1471-2105-9-64 (PMC2253513; doi:10.1186/1471-2105-9-64)
Supplement: Additional file 6 — Topological shape of a cluster and its functional annotations. Cluster 22 in Additional File 1. (a) sub graph of Cluster 22 extracted from DIP PPI network. Each protein is annotated by MIPS functional category. (b) MIPS functional IDs and their corresponding literal names. The best accordant functional term is boldfaced. [file 1471-2105-9-64-S6.pdf]

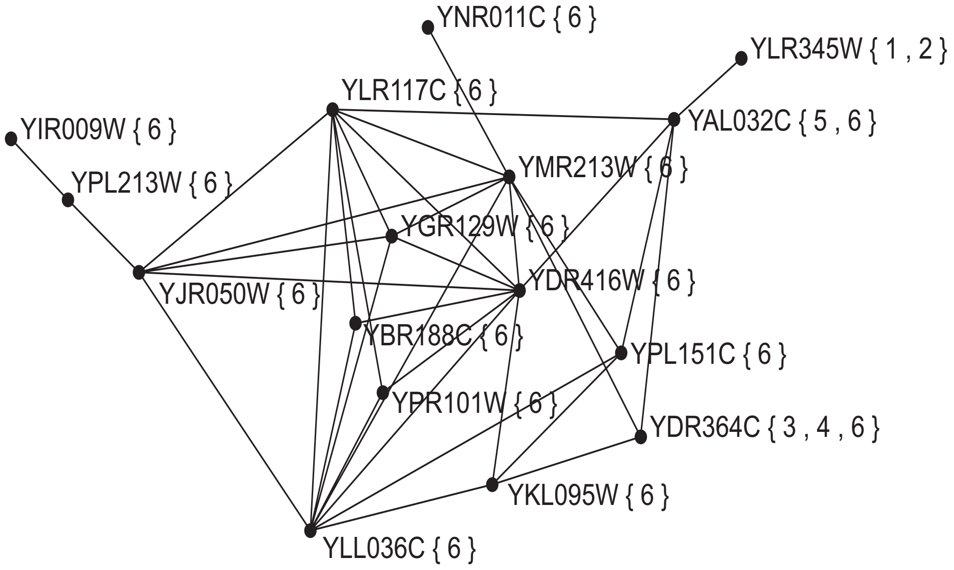

(a)

Function ID	MIPS ID	Function name
1	01.05.25	regulation of C-compound and carbohydrate metabolism
2	02.01	glycolysis and gluconeogenesis
3	10.03.01.01.05	S phase of mitotic cell cycle
4	10.03.05.01	spindle pole body/centrosome and microtubule cycle
5	11.02.03.04.01	transcriptional activator
**6**	**11.04.03.01**	**splicing**

(b)

Fig. 4: Topological shape and functional annotations of Cluster 22 in Additional file 1. (a) sub graph of Cluster 22 extracted from DIP PPI network. Each protein is annotated by MIPS functional category. (b) MIPS functional IDs and their corresponding literal names. The best assigned functional term is boldfaced.
