# Supplementary material for: CASCADE: a novel quasi all paths-based network analysis algorithm for clustering biological interactions
Source: BMC Bioinformatics. 2008 Jan 29;9:64. doi: 10.1186/1471-2105-9-64 (PMC2253513; doi:10.1186/1471-2105-9-64)
Supplement: Additional file 7 — Topological shape of a cluster and its functional annotations. Cluster 25 in Additional File 1. (a) sub graph of Cluster 25 extracted from DIP PPI network. Each protein is annotated by MIPS functional category. (b) MIPS functional IDs and their corresponding literal names. The best accordant functional term is boldfaced. [file 1471-2105-9-64-S7.pdf]

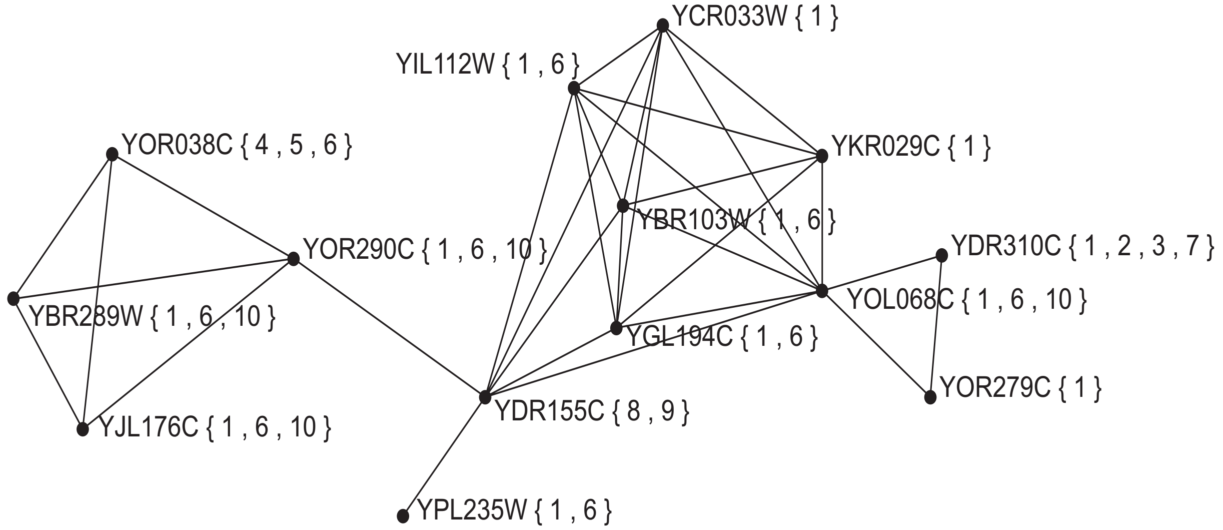

(a)

Function ID	MIPS ID	Function name
**1**	**10.01.09.05**	**DNA conformation modification** (**e.g. chromatin**)
2	10.03.01.01	mitotic cell cycle
3	10.03.01.03	cell cycle checkpoints (checkpoints of morphogenesis, DNA-damage,-rep., mitotic phase and spindle)
4	10.03.04.01	centromere/kinetochore complex maturation
5	11.02.03.01	general transcription activities
6	11.02.03.04	transcriptional control
7	11.02.03.04.03	transcriptional repressor
8	14.01	protein folding and stabilization
9	32.01	stress response
10	34.11.03.07	pheromone response, mating-type determination, sex-specific proteins

(b)

Fig. 5: Topological shape and functional annotations of Cluster 25 in Additional file 1. (a) sub graph of Cluster 25 extracted from DIP PPI network. Each protein is annotated by MIPS functional category. (b) MIPS functional IDs and their corresponding literal names. The best assigned functional term is boldfaced.
